# First-time postpartum motherhood during armed conflict: experiences, challenges, coping, and support needs among women with mobilized partners

**DOI:** 10.3389/fgwh.2026.1774107

**Published:** 2026-07-16

**Authors:** Yifat Findling, Ilana Chertok, Keren Dopelt

**Affiliations:** 1 School of Health Sciences, Ashkelon Academic College, Ashkelon, Israel; 2 School of Nursing, Ohio University, Athens, OH, United States

**Keywords:** armed conflict, coping, first-time mothers, postpartum period, resilience, transactional model of stress and coping, trauma-informed postpartum care

## Abstract

Armed conflict creates profound challenges for first-time mothers navigating the early postpartum period without the presence of their partners. The abrupt loss of expected support, combined with heightened fear and prolonged uncertainty, intensifies emotional vulnerability and disrupts maternal adjustment to early parenthood. Despite extensive research on trauma and crisis, little is known about how large-scale armed conflict shapes first-time mothers' lived postpartum experiences and their engagement with care systems. This study aimed to explore these experiences among women who gave birth during an armed conflict while coping alone with infant care due to their partners' military deployment. Twenty first-time mothers participated in this qualitative study, which included in-depth, semi-structured interviews. Data were analyzed using thematic analysis and interpreted through Lazarus and Folkman's Transactional Model of Stress and Coping, following COREQ guidelines. Four themes emerged: (1) Forced single parenthood, (2) The complex role of first-time motherhood in war, (3) Fears and anxieties, and (4) Coping strategies and resources. Participants described emotional exhaustion, fear, loneliness, disrupted bonding, and heightened alertness. A recurrent finding was the clear gap between mothers' expectations for compassionate, continuous postpartum support and the task-oriented, limited emotional care they received from professionals. Mothers relied on internal resilience, family support, community assistance, and breastfeeding as coping mechanisms. These findings demonstrate how armed conflict intensifies maternal vulnerability and reshapes postpartum experiences, underscoring the need for trauma-informed, relational postpartum care and stronger continuity between community and hospital services for mothers navigating early parenthood in conflict settings.

## Introduction

War is a source of stress, and its impact often extends beyond the direct victims, creating an overarching sense of danger, anxiety, and psychological distress among the broader population (1, 2). Wartime conditions create unprecedented challenges for new mothers. Emotional instability, uncertainty, and physical fatigue are amplified by isolation and a sense of abandonment (1, 3). Given that the postpartum period is already a vulnerable time requiring physical recovery and emotional adjustment, wartime instability may further intensify these stressors and hinder access to essential supportive care. To address these needs, nurses and midwives play a central role in promoting maternal well-being and recovery through continuity and compassionate postpartum care (4–6). Despite increasing evidence on the psychological impact of armed conflict on civilian populations, limited research has explored the postpartum experiences of first-time mothers during wartime conditions. This gap underscores the need for a more comprehensive understanding of their specific challenges and the professional responses necessary in crisis settings.

The stress during wartime may increase the risk of parental burnout arising from a chronic imbalance between high parenting demands and limited coping (7). Additionally, the lack of support from a partner due to military service may exacerbate mothers’ parental burnout (8, 9). Furthermore, wartime conditions, including siren warnings of threatened attacks and the need to seek shelter throughout the day and night, are disruptive to new mothers' postpartum recovery and function, including sleep and breastfeeding (10, 11). The present study is situated in Israel during the Iron Swords War, which began on October 7, 2023, and continued to shape civilian life throughout the study period.

According to Lazarus and Folkman's (12) Transactional Model of Stress and Coping, individuals appraise stressful situations through two key cognitive processes: primary appraisal, in which the event is evaluated as threatening, harmful, or challenging; and secondary appraisal, in which the available resources to cope with the stressor are assessed. This theoretical model provided an adequate conceptual framework for understanding how mothers interpret and respond to the challenges of first-time motherhood during wartime. However, parenting in such conditions heightens stress levels, as parents must navigate child-rearing alongside constant threats and uncertainty (13, 14). The dual burden of first-time motherhood and wartime anxiety intensifies the need for adaptive coping mechanisms, illustrating the model's relevance in capturing the dynamic interplay between stress appraisal and coping in extreme familial and sociopolitical contexts. Understanding and explaining these experiences is important not only for documenting mothers' distress during wartime, but also for identifying how maternal care systems can respond more effectively to their emotional, relational, and practical needs. Therefore, the aim of the present study is to explore the lived experiences and challenges of first-time mothers in Israel who gave birth during the Iron Sword War, while their partners were mobilized or absent from home for extended periods due to military or security-force service. By capturing the intersection of maternal vulnerability, emotional coping, and wartime disruption in order to inform postpartum care practices and support strategies for mothers navigating early parenthood in conflict settings.

## The study

Previous studies have documented the impact of partner absence on maternal well-being in diverse contexts of extreme stress, such as migration, incarceration, or long-term military deployment (15, 16). These situations, though distinct from active war, share common features of emotional overload, increased caregiving demands, and disrupted couple dynamics. Research has shown that the lack of spousal support during the postpartum period may heighten maternal anxiety, impair bonding, and limit coping resources (17, 18).

## Methods

### Study context

The study was conducted in Israel between March and June 2025, during the ongoing armed conflict that began on October 7, 2023. During this period, civilian communities, particularly in northern and southern Israel, were exposed to repeated security threats, including sirens, missile and rocket alerts, and the need to move quickly to protected rooms or public shelters. Some families were evacuated from their homes, while others temporarily relocated to relatives or alternative housing arrangements to increase safety and obtain support.

The conflict also involved extensive mobilization of reserve and security forces. Accordingly, the present study focused on first-time mothers whose partners were absent from home for extended periods due to military or security-force service during pregnancy, childbirth, or the early postpartum period. This context provides the background for examining postpartum experiences among first-time mothers caring for an infant during armed conflict and partner absence.

### Design

The study design employed an interpretive qualitative approach, utilizing semi-structured in-depth online interviews and the collection of non-identifiable sociodemographic data. The interpretive approach enabled the exploration of participants' lived experiences and the meanings they ascribe to them. To interpret these experiences, Lazarus and Folkman's Transactional Model of Stress and Coping (12) was employed as a theoretical lens, allowing for a deeper understanding of how participants appraised and responded to the challenges described.

This qualitative study followed the COREQ guidelines and included 20 first-time mothers who gave birth during the war. Semi-structured interviews explored mothers' emotional and practical experiences of postpartum life amid ongoing conflict and partner absence. A thematic analysis approach was used to identify recurrent patterns related to coping, professional and social support, and systemic responses within maternal care. The analysis was conducted using an inductive approach, with themes developed from the data before being considered in relation to the theoretical model. This design enabled an in-depth exploration of mothers' meaning-making processes in conditions of armed conflict. The Transactional Model of Stress and Coping served as a sensitizing framework, guiding but not constraining the analysis. Finally, no software program (e.g., NVivo or ATLAS.ti) was used; coding was conducted manually using iterative line-by-line analysis.

### Participants

The participants chosen for the research were 20 mothers residing in a conflict-affected region who gave birth to their firstborn child during the war and whose partners were absent from home for extended periods due to active military or security forces duty. The mothers were recruited through advertisements in social media groups for pregnant and postpartum women by the principal investigator, as well as through a snowball sampling method. Eligibility criteria included being a first-time mother, giving birth during the ongoing armed conflict, experiencing partner absence due to military mobilization, and being over 18 years of age. The interviews were conducted online or via telephone, according to each mother's preference, between March and June 2025, with an average interview duration of 60 min. Sample adequacy was determined according to the concept of information power (19), whereby the specificity of the sample, homogeneity of participants, and richness of the interview data were sufficient to address the study aim.

### Researcher positionality and reflexivity

The interviews were conducted by a maternal-infant health nurse experienced in postpartum and community care. This background may have influenced interview dynamics and interpretation. Reflexive notes were kept to acknowledge and reduce potential bias. The researcher engaged in ongoing reflexive questioning throughout data collection and analysis to increase transparency and enhance the trustworthiness of the findings.

### Tools

The interview guide was designed to support an open exploration of mothers' postpartum experiences during wartime. A semi-structured interview guide was developed by the researchers based on the relevant research literature. Topics included responding to infant needs and establishing bonding in conflict areas (20); breastfeeding support through social networks (21); breastfeeding experience during wartime (10); maternal emotions, maternal sleep, and infant sleep (11); parental burnout (22, 23); coparenting challenges and coping strategies among military couples (24); and community-based support and resilience building (25). The interview guide was pilot-tested with two mothers to ensure clarity and relevance, with minor adjustments made prior to data collection.‏

The mothers were asked to describe experiences, emotions, thoughts, and behaviors with the aim of deepening the understanding of the experience of motherhood to the first child who was born during wartime, while their partners were drafted and absent from home for an extended period. The mothers were asked about their pregnancy, birth, postpartum, and motherhood experience and feelings while coping with daily challenges in caring alone for their infant in the absence of their partner, who actively served. The mothers were also asked about the involvement of healthcare staff, family, friends, and community in assisting with the parenting role and responsibilities during the war. The interview guide is provided as Supplementary Material, in accordance with Frontiers guidelines.

### Data analysis

The interviews were conducted in the participants' native language by a qualified nurse researcher with expertise in maternal-infant health. The interviews were recorded and transcribed. All transcripts were anonymized immediately after transcription and stored on a secure, password-protected server. The transcripts were deidentified and reviewed multiple times by the researchers to develop a codebook. The interviews were analyzed using a thematic analysis method guided by Lazarus and Folkman's (12) Transactional Model of Stress and Coping. The thematic analysis of the interviews was conducted in several stages, following Shkedi's (26) method. In the first stage, the researchers read all the interviews to familiarize themselves with the data. In the next step, ideas, categories, and themes related to the research questions were identified by each reader. The differences between readers were addressed through team discussions, during which alternative interpretations were explored and reflected upon. Each researcher contributed their perspective, and these discussions were used to refine the coding framework, deepen interpretation, and strengthen the thematic structure. Rather than seeking inter-coder agreement or a single correct interpretation, the discussions served as part of the interpretive analytic process. After the thematic structure had been refined and finalized, representative quotations were selected to illustrate the themes. The Transactional Model of Stress and Coping (12) was used as a theoretical lens to guide the interpretation of the qualitative data. The model served to orient the thematic analysis and conceptualize mothers' experiences of stress appraisal and coping during wartime, consistent with the interpretive design of the study. The themes and quotes were translated and documented in English at the final stage. Translation was conducted using a forward-translation process by the researcher, followed by a review for accuracy and meaning preservation. We used a standardized codebook to ensure the validity of the translations from the original language to English. Validation of the analysis was strengthened through peer debriefing (27) and through maintaining an audit trail documenting analytic decisions throughout the process. Validation of the thematic structure of the findings was conducted through a peer debriefing process (27), which continued until an agreement was reached on the classification method (28).

### Study rigor

The study was approved by the Institutional Ethics Committee. Before the interview, the mothers were asked to sign a consent form that detailed the purpose of the study, their right to stop the interview at any stage, confidentiality, and their consent to be recorded during the interview. The trustworthiness of the findings was ensured according to Lincoln and Guba's criteria (27). Credibility was supported through repeated engagement with the transcripts, collaborative analytic discussions among the research team, peer debriefing, and grounding the themes in participants' accounts. Differences in interpretation were discussed as part of the analytic process and used to refine the thematic structure, rather than to establish inter-coder reliability. Transferability was enhanced by providing a detailed description of participants and their wartime contexts. Dependability was supported by maintaining a clear audit trail of data collection and analysis. Confirmability was established by grounding interpretations in participants' quotes and maintaining reflexive notes. Authenticity was ensured by allowing mothers to express their experiences in their own words. Analytic transparency was supported through peer debriefing, reflexive notes, and an audit trail documenting key analytic decisions. The study followed the COREQ checklist (29) to ensure transparency and rigor throughout the research process. Data were stored securely on a password-protected server in accordance with ethical requirements. These strategies ensured methodological transparency and strengthened the trustworthiness of the findings, aligning with the standards commonly expected in qualitative health research.

### Ethical considerations

The study was approved by an institutional ethics committee (specific institutional details are omitted to maintain anonymity for peer review). All participants received an explanation of the study and provided written informed consent. Given the potentially distressing nature of the interviews, participant safeguarding procedures were implemented. Participants were informed that they could pause, skip questions, or stop the interview at any time without providing an explanation. The interviewer, a maternal-infant health nurse, monitored signs of emotional distress during the interview and offered pauses when needed. At the end of the interview, participants were asked how they felt and reminded of available sources of emotional support, including their health maintenance organization, maternal-infant clinic, family physician, and community mental health services. Participants who described acute emotional distress or safety concerns were encouraged to seek appropriate professional support. All identifying information was removed from transcripts in accordance with ethical requirements. The study was conducted in accordance with the principles of the Declaration of Helsinki.

### Findings

The findings are presented according to the themes identified through the thematic analysis. Descriptive characteristics were collected to contextualize participants' postpartum experiences.‏

Twenty mothers of infants born during the war participated in the interviews. The mother's mean age was 27.5) *SD* = 2.28, range 22–31) years. Most mothers have an academic degree (Bachelor's *n* = 10, Master's *n* = 4) 2 graduated high school and 4 were student.

The children's ages ranged from 6 to 18 months. As a result of the war, 30% were evacuated from their homes, and 55% temporarily relocated to live with relatives.‏ Approximately half of the interviewees (45%) sought emotional, couples, or parenting guidance support. The characteristics of mothers are presented in Table 1.

**Table 1:** Characteristics of participating mothers who gave birth during the war (*N* = 20).

Characteristic	Category	*n*
Age of mothers (years)	Mean (SD)	27.5 (2.28)
	Range	22–31
Educational level	High school graduate	2
	Bachelor's degree	10
	Master's degree	4
	Student	4
Living arrangement during the war/postpartum period	Evacuated to a hotel	5
	Temporarily lived with parents or relatives	11
	Other temporary support arrangements	4
Infant age at interview	6–9 months	4
	10–14 months	3
	15–18 months	13
Breastfeeding status	Ongoing breastfeeding	14
	Started and stopped breastfeeding	3
	Did not breastfeed	3
Support seeking	Sought emotional/couple/parenting therapy	9
	Did not seek formal support	11

To strengthen the integration of the Transactional Model of Stress and Coping, the findings are presented not only as thematic categories but also as interpretive accounts of how participants appraised wartime postpartum conditions and mobilized coping resources. The first three themes broadly illustrate mothers' primary appraisal of threat, loss, and role disruption, whereas the fourth theme illustrates secondary appraisal and coping, including the identification, use, and limits of internal, relational, professional, and community resources. The model was therefore used as an interpretive lens to understand how mothers moved between perceived threat, perceived resource availability, and coping actions.

The thematic analysis revealed four themes, each with subthemes according to the Transactional Model of Stress and Coping (12), (Table 2): 1. Forced single parenthood within a relationship: (a) Shattered dreams, (b) Loneliness and helplessness, (c) Survival parenting, (d) Self-blame; 2. Complex role of motherhood in war: (a) Maternal burnout vs. empowerment, (b) Role reversal, (c) Success/failure dichotomy, (d) Lack of emotional bonding and depression vs. the desire to feel normal; 3. Fears and Anxieties: (a) Anxious and overprotective mothering, (b) Fear for the father, (c) War-triggered nightmares; 4. Coping strategies and resources: (a) Inner strength and resilience, (b) Internal: Motherhood role, (c) External: Support network.

**Table 2:** Themes and subthemes according to the transactional model of stress and coping (12).

Transactional Model of Stress and Coping stages	Themes	Subthemes	Interpretive link to the Transactional Model
Stress Appraisal	Forced single parenthood	Shattered dream	Partner absence was appraised not only as a practical caregiving difficulty, but as a loss of expected shared parenthood and a threat to emotional safety, maternal identity, and family continuity.
		Loneliness and helplessness	
		Survival parenting	
		Self-blame	
	The complex role of motherhood in war	Maternal burnout vs. empowerment	Mothers appraised the combined demands of infant care, partner absence, and wartime uncertainty as overwhelming and, at times, exceeding their available emotional and practical resources. Some accounts also reflected reappraisal, as mothers gradually recognized endurance, competence, and maternal agency.
		Role reversal	
		Success vs. failure	
		Lack of emotional bonding vs. the desire to feel normal	
	Fears and Anxieties	Anxious and overprotective mothering	Sirens, shelter access, concern for the partner's survival, and fears about the father–infant bond repeatedly activated perceptions of danger, shaping daily decisions about mobility, sleep, care-seeking, and exposure to news.
		Worries about the spouse: As a father, as a husband	
		War-triggered nightmares	
Coping strategies and resources		Inner strength and resilience: Emotional regulation and emotional work News and media	Mothers evaluated and mobilized internal coping resources, including emotional regulation, self-reliance, limiting exposure to distressing news, and attempts to maintain functioning despite fear and exhaustion.
		Internal resources: Motherhood role Love and care for the infant Breastfeeding as a source of resilience, bond, and anchor	The motherhood role and breastfeeding functioned as emotionally meaningful resources that helped some mothers regulate distress, maintain a sense of purpose, and strengthen mother–infant connection.
		External resources: Support networks The gap between desired and received support Partner Family support Community, friends’ support, and social media support groups Professional support: Mixed experiences	External resources were appraised according to their availability, reliability, and emotional usefulness. Support from partners, family, peers, community, and professionals could buffer distress, whereas absent, inconsistent, or task-oriented support intensified feelings of isolation and overload.

### Forced single parenthood

The first theme concerned the mothers' experience of unplanned or “forced” single parenthood due to the war, following their partners' military mobilization. The challenges of first-time motherhood were intensified by the need to care for a newborn alone. This theme was reflected in the following subthemes: Shattered dreams, loneliness and helplessness, survival parenting, and self-blame.

#### Subtheme 1. Shattered dreams

During pregnancy, mothers envisioned joyfully sharing parenting. Due to the war, these dreams were shattered, leaving them to parent alone. The gap between expectation and reality gave rise to frustration, sorrow, and a sense of loss, not only for their own unfulfilled hopes but also for their partners' lost opportunity to experience fatherhood. “*I had planned for this period to be different, to go out with the infant… to spend time together as a family. It was very hard for me to accept that the situation didn’t allow for that. I felt sad, because my husband had waited his whole life to become a father; it was truly his dream. And suddenly, he has an infant, and he's not even at home”* [12].

“What was hardest for me was not being able to share with my husband what I was going through. I kept thinking, how can it be that I'm a single mother? I never chose this” [9].

These disruptions extended beyond the emotional bond with the partner to the entire framework the mothers had imagined for their postpartum experience. Several mothers shared plans they had envisioned, now rendered unattainable. “*I had imagined life after childbirth to be entirely different. I had a very clear and organized plan: That my husband and I would care for our infant together, that I would continue my studies while he supported and assisted me, that we would remain within our beloved community, and that we would lead a simple and peaceful life”* [18].

As these expectations collapsed, mothers reported intense emotional responses. Feelings of grief, helplessness, and anger were common, underscoring the emotional toll of this unexpected shift to solo parenting. “*I felt as though my entire life had turned upside down. I cried a lot and was filled with frustration and anger”* [8].

#### Subtheme 2. Loneliness and helplessness

The mothers expressed a sense of loneliness and helplessness as they managed alone, compounded by the inability to communicate with their partners, who were called to military service. They revealed an urgent need for connection, support, and assistance physically but also emotionally, especially from their partners, even when getting support from family and friends. “*I felt extremely lonely … I couldn't cope without my husband, who was so far away and unreachable, and with whom I had no ability to speak or share what I was going through”* [8].

The separation from their partners caused mothers to feel vulnerable and overwhelmed, although they often had to demonstrate composure and self-reliance in front of others. “*I was very sad, with a terrible sense of loneliness and helplessness, and a deep frustration that I didn't have a partner, as I had hoped, to help care for the infant. I cried a lot, and I'm certain that if I had given birth at a different time, with my husband by my side, everything would have looked different. I would have felt more secure, I would have relied on him, consulted with him, felt comfortable being vulnerable around him, unlike when I am with my parents, when I feel like I must stay strong and not fall apart”* [20].

#### Subtheme 3. Survival parenting

Several mothers described entering a state of emotional numbness, referring to themselves as being on “auto-pilot.” In this mode, they primarily focused on the technical aspects of infant care, while feeling emotionally disconnected from their infants. This disconnection generated a sense of guilt and internal conflict, as it clashed with their pre-existing expectations of first-time motherhood. “*During the first six months, I was like a robot. I simply managed the situation. The infant received everything he needed; he ate on time, bathed, slept, got his vaccinations, and was monitored for weight. He got everything from me except love. When he cried, I cried with him. I felt he was too much for me to handle, and it was very difficult for me to accept that this was the kind of mother I had become, a mother who performed only technical care, a mother disconnected from her infant, who felt no affection or love toward him, a mother who saw her own infant as a burden”* [14].

This sense of mechanical caregiving was often linked to physical and emotional exhaustion, and to the worry and fear related to war and their partners' absences. Mothers describe going through the motions of daily care while feeling detached and emotionally unavailable. “*In the beginning, I was utterly exhausted. I barely slept; I was deeply worried about my husband at war and about the situation in the country. I operated on autopilot; everything was mechanical. I did everything that needed to be done, but I didn't enjoy any of it. I felt that I wasn't giving my infant what she really needed beyond food and bathing. I wasn't truly present. I was preoccupied with existential anxiety and couldn't be emotionally available to truly experience motherhood”* [1].

Along with exhaustion and disconnection, many mothers were aware of the gap between their long-standing desire for motherhood and the difficult reality they encountered. “*I felt guilty toward my infant, as I had waited for him for so long, yet suddenly found the care for him to be exhausting”* [8].

#### Subtheme 4. Self-blame

The mothers' sense of guilt was often intertwined with self-blame, as they struggled to reconcile their limitations with their ideals of motherhood. Facing the demands of infant care alone, many felt they were falling short, lacking patience, warmth, or the emotional availability they believed a “good mother” should embody. “*There were days when I was completely out of patience, I would tell my infant to go to sleep, ask why she wasn't sleeping, why she was crying… I would actually speak to her with frustration. Then I would feel terribly guilty and regretful, because after all, she's just an infant, and it's not her fault that I'm doing this alone, that I'm exhausted and don't have the strength to care for her. The situation really pushed me to the edge”* [10].

Mothers experienced acute dissonance during moments that should have brought joy, such as witnessing the *infant*'s developmental milestones, which instead triggered sadness, fatigue, or loneliness. “*From time to time, I had breakdowns. I felt guilty when I was extremely tired and my infant did something new, and I didn't have the energy to be happy about it. I had no partner to witness it with me, to experience it here and now, in real time”* [9].

Interpreted through the Transactional Model, forced single parenthood represented a primary appraisal of loss and threat. Mothers did not describe partner absence only as a practical difficulty, but as a disruption of the expected transition to parenthood, the imagined shared family unit, and their sense of emotional safety. The repeated contrast between “what should have been” and “what actually happened” demonstrates how wartime partner absence was appraised as a threat to maternal identity, family continuity, and postpartum adjustment.

### Complex role of first-time motherhood in war

As partners were mobilized to military duty for extended periods during the war, the mothers' roles became more complex as they had to take on more responsibilities themselves. Within the context of the war and the prolonged absence of their partners, mothers needed to create a sense of safety and calm amid the chaos. Mothers navigated between their loyalty to their family and to the nation. As the sole parental figures who were physically and emotionally present with the children, the mothers were responsible for their infants’ constant care and for shielding them from the threats of war. This maternal identity was intense, as the mothers felt they had to maintain the family's stability. The constant demands on their time and attention contributed to maternal burnout, a sense of failure, lack of emotional bonding, and depression.

#### Subtheme 1. Maternal burnout vs. empowerment

First-time motherhood was described as both exhausting and a source of inner strength and empowerment. While some mothers reported extreme fatigue, self-dissatisfaction, and emotional volatility, others described moments of personal growth, psychological resilience, and pride in their ability to manage alone in the absence of their partner. This duality reflects the tension between vulnerability and self-discovery that characterized their maternal journey. “*I feel there were times when I am burned out, physically exhausted, and not always satisfied with my maternal functioning. I learned that my emotional range is very broad and that I can quickly shift to negative emotions. I know that I must act in my own interest and that of my infant, to become a mentally stronger mother”* [6].

Another mother described how, despite anticipation that her partner's return would bring relief, she recognized her own agency in coping and adapting. “*I felt truly burned out as a mother. I was so exhausted that regardless of how much sleep I managed to get, the fatigue never ended. I constantly told myself that the solution would come when my husband returned, but suddenly I realized I had lifted myself up not because he returned from war, but through my own merit and the decisions I made”* [8].

#### Subtheme 2. Role reversal

A pattern of role reversal emerged when mothers, having assumed sole responsibility for infant care during their partners' military service, also became caregivers to physically or emotionally wounded husbands upon their return. This reversal, sometimes occurring during pregnancy, birth, or postpartum, left some mothers feeling invisible, emotionally unsupported, and unable to access the care they themselves needed. “*My husband was in active duty and was wounded. I was ∼40 weeks pregnant, and instead of being cared for as in any normal situation when a woman is nearing birth, I found myself caring for him, staying by his side around the clock, helping him shower and dress. Instead of having a happy and supportive husband by my side and having childbirth be a positive and emotional experience, I found myself worrying about his well-being while giving birth* [3].

The emotional burden of tending to both an infant and a traumatized or wounded partner, with limited support, was described by several mothers as overwhelming. Some felt sidelined during their own postpartum experience and were expected to provide care and support without receiving any in return. “*I was concerned that my husband might commit suicide due to everything he experienced, so it was important for me to be at home rather than at my parents’. On the other hand, I constantly thought about myself and what I was going through. It was very difficult because I found myself caring for my wounded husband and caring for my infant, while no one saw me. Everyone was occupied with him or the child, and I, a postpartum mother … became invisible.”* [6].

#### Subtheme 3. Success vs. failure

The mothers articulated a dynamic tension between competing self-conceptions of “good” vs. “poor” motherhood. Periods of household dysfunction were perceived as evidence of maternal failure, whereas moments of overcoming adversity were framed as confirmation of maternal competence and success. Strategies for achieving a sense of maternal success included setting simple, attainable goals, deliberately minimizing emotional involvement, and reducing exposure to distressing external stimuli such as news coverage of the war. “*I felt everything was new to me … I am a first-time mother during war-time with my husband deployed. I constantly cried. I focused only on simple things, giving less emotional attention to the infant. Over time, my emotional capacity improved; I learned that I am a good and sensitive mother and that I am mentally stronger.”* [11].

Another mother described a similar trajectory, from initial functional struggle to a retrospective sense of achievement and maternal adequacy: “*I think that initially, functionally, it was difficult for me. I was very preoccupied with the country's situation, watching the news rather than truly being with the infant. Today I am very satisfied with my functioning and believe I did good work. I have a happy child, not a ‘war child,’ and I am a good mother, truly good.”* [12].

#### Subtheme 4. Lack of emotional bonding and depression vs. the desire to feel normal

Several mothers described difficulty forming an emotional bond with their infants, alongside a deep yearning to experience a sense of normalcy in first-time motherhood. Feelings of sadness, anxiety, and emotional withdrawal often created internal conflict between how they felt and how they believed they should feel due to personal or societal maternal expectations fueled self-doubt and inadequacy. “*I felt great anxiety … especially when the infant cried and nothing worked to calm him. I felt terrible that I couldn't understand how to help the infant when he cried, thinking that only I was experiencing this and that other mothers might have it easier. I felt … that something about me was probably abnormal and wrong”* [2].

For some mothers, this emotional disconnect was accompanied by a pervasive sense of entrapment, of going through the motions of infant care while feeling that their own identity had been eclipsed. “*It was a terrible period. I had a real nightmare feeling that I was out of control of my life … I felt I had no life … I felt trapped.”* [18].

This theme demonstrates how mothers' appraisal of the situation shifted over time. Initially, the combined demands of infant care, partner absence, and wartime uncertainty were appraised as overwhelming and as exceeding available resources. However, some mothers later reappraised their own capacity, describing competence, endurance, and maternal agency. This movement from perceived inadequacy to perceived capability illustrates the dynamic nature of appraisal and coping in the Transactional Model.

### Fears and anxieties

The mothers described a profound and pervasive sense of fear while raising their infants during war-time. Their concerns encompassed multiple layers of threat: Physical safety, the absence and vulnerability of their partners in active duty, and that the father-infant bond might not form. These fears were intensified by the constant threat of rocket attacks and by the looming fear of losing their partner in the war. These overlapping fears intruded upon daily life, disrupted sleep, and heightened anxiety to the point of emotional and functional impairment. For many mothers, fear left little room for emotional availability, joy, or self-care, thereby compounding the challenges of first-time motherhood in times of crisis.

#### Subtheme 1*.* Anxious and overprotective mothering

The mothers expressed profound distress and fear related to bringing a child into a world marked by war and chaos. The joy and anticipation that typically accompany childbirth were, for many, overshadowed by concerns about their infant's safety, the overall instability, and their own feelings of uncertainty to cope with the dual demands of first-time motherhood and war-time anxiety. “*It was a very difficult time emotionally. I gave birth during the war and couldn't connect with my infant. Thoughts ran through my head like, ‘What kind of world did I bring her into?’ and ‘What have I done to her, bringing her into such a chaotic and destructive reality’”* [11].

Fears related to the war affected mothers' behaviors and routines. Many avoided leaving their homes, even for essential appointments, out of fear of rocket attacks. “*There were many sirens because of rockets fired toward our area, and I was really scared. I feared for myself and for my baby girl, and I didn't leave the house. We discovered she had ankyloglossia (tongue-tie) … I took her to an otolaryngologist doctor, during blaring sirens and fear”* [7].

Others recounted how specific scenarios, such as being alone in the car with their infants during a siren, led them to postpone or avoid seeking care altogether. “*At first, I didn't go to the maternal-infant clinic. I was afraid of the situation. I was scared that a siren would go off while I was on the way, and I was alone with the infant. What would I do? How would I get him out of the car and lie down with him on the ground?”* [5].

Some mothers took active measures to reduce their exposure to risk by temporarily relocating to homes of relatives with better physical protection. “*I went to my parents’ house, not only because I didn't want to be alone, but also because our apartment doesn't have a safe room. There's a shelter in the neighborhood, but it takes time to get there when there are sirens. I was afraid for myself and for my infant, so I alternated between staying at home and at my parents’”* [13].

#### Subtheme 2. Fear for the father

Many mothers expressed a constant looming fear that their partners might not return from war. The dread of the “knock on the door,” symbolizing military death notification, was deeply embedded in the mothers' consciousness and often colored their perceptions of their future. These fears compounded the mothers' concerns about fathers' prolonged absences during the war and the potential impact on the father-infant bond. “*I was so afraid for my husband's safety, especially after one of his friends was killed near the area he was in. My biggest fear was the ‘knock on the door.’ There was a point when I asked my friends to call me before they came over, just so I wouldn't be startled by someone knocking. It shook me that deeply”* [17].

“What I feared most was the ‘knock on the door.’ I would imagine hearing a knock and immediately spiral into stress, unable to sleep. So many thoughts ran through my head. I wondered how I would react, what I would do alone, what my life would look like, how everything would change in that moment” [3].

Many feared that the formative early months of life, important for attachment, would pass without the father's involvement, jeopardizing their infant's emotional development and the partner's sense of parental connection. In some cases, such interpretations may have reflected the mother's own fears, raising the possibility of emotional projection, as she tried to understand her infant's behavior.

“I was really afraid that no bond would form between him and our infant. But the bond that developed was actually good; he made a real effort to be present with her every time he came home, even if only for a short time” [7]

While some mothers were reassured by the quality of interactions during brief reunions, others noticed subtle signs of insecurity or confusion in their infants, which they attributed to the father's intermittent presence. “*Even though I was worried the bond would be weak because he didn't see him much … every time he came home, he was really present, and maybe that helped. But what did happen was that as the infant grew, sometimes he wouldn't want his father to put him to sleep. I felt as if he didn't want to fall asleep because he wasn't sure his dad would be there when he woke up, like he couldn't trust that his dad would be around in the morning, so he simply refused to go to sleep.”* [12].

#### Subtheme 3. War-triggered nightmares

The mothers described severe sleep disturbances and war-induced nightmares that impaired their daily functioning. The combination of fear of sirens, lack of access to shelters, and absence of their partners created a constant sense of vulnerability, preventing them from deep and restorative sleep. Nights were marked by hypervigilance, exhaustion, and intrusive thoughts, all of which intensified emotional distress during an already fragile postpartum period. “*Sleep was a major issue for us. When the war began, it was accompanied by real nightmares, and I couldn't function. The infant slept next to me in a co-sleeper. He would fall asleep only through nursing and only in my arms … I was alone; my husband wasn't with me to help. I was completely exhausted. I felt like I was on the verge of collapse, like I was about to lose my sanity”* [19].

For many mothers, the inability to rest stemmed from infant care demands and the ever-present fear of danger. Some mothers deliberately limited their sleep out of fear that they would not wake up in time to protect their *infant* in the event of a rocket attack. “*I slept in the same room with her, with her crib next to my bed. I had a lot of nightmares at night and couldn't feel at ease. I was worried about my husband, worried about my daughter, afraid something might happen to us. There were sirens part of the time, and we don't have a safe room, only a neighborhood shelter. I was extremely stressed about whether I'd be able to reach the shelter in time and whether I'd even wake up during the night. So, I only allowed myself to sleep lightly”* [11].

The fears described by participants reflect an ongoing process of threat appraisal rather than a single discrete stressor. Sirens, shelter access, concern for the partner's survival, and fears about the father–infant bond repeatedly activated mothers' perception of danger. These appraisals shaped daily decisions, including whether to leave the house, seek care, sleep deeply, consume news, or remain close to protected spaces.

### Coping strategies and resources

The mothers described drawing on both internal and external resources to navigate the challenges of first-time motherhood during war-time. Internally, they relied on self-discipline and a deep sense of responsibility toward their infants. Many emphasized their emotional commitment to their infant as a motivating force that helped them persevere despite exhaustion, fear, and uncertainty. Breastfeeding was described as a physical necessity and a source of emotional connection and comfort for the mother and her infant.

#### Subtheme 1*.* Inner strength and resilience

Amid the pressures of war-time motherhood, many mothers discovered unexpected inner strength and resilience. Despite fear and exhaustion, they found ways to function, protect their infants, and maintain a sense of control. The mothers developed practical and emotional coping strategies to manage the demands of first-time motherhood during war-time. They reported increased self-reliance alongside openness in seeking and accepting help. “*This period made me realize that I'm capable of anything, no matter how hard it gets. I learned to adapt and manage everything on my own. I also learned to trust others with my infant and understood it's okay to ask for and receive help. I'm a caring, attentive mother who's always there for her infant. I've become more capable and confident that anything is possible if I believe in it”* [10].

For some, the experience of “surviving everything” became a source of emotional stability in itself, proof of their capacity to cope under extreme conditions. “*I discovered strengths I didn't know I had. I managed to survive everything and realized I have resilience and abilities. I don't need anyone, I can function despite everything, and that thought calms me”.* [14].

To protect their infants from stress, mothers actively managed their emotions, often suppressing fear, sadness, and anxiety. Several described “donning a mask”, deliberately performing calmness and emotional stability even when feeling overwhelmed, enacting expected emotions rather than expressing authentic ones. This form of surface acting allowed them to create a sense of safety for their infant, even as they internally struggled with emotional distress. “*I know how to regulate my emotions when I'm with my infant so she feels safe and isn't exposed to my stress. I started therapy, which helped me, especially in managing emotions when I'm overwhelmed and alone”.* [17].

Exposure to war-related news often intensified anxiety and disrupted mothers' functioning. While some chose to disconnect entirely from media to preserve their well-being, others felt compulsively drawn to updates, despite recognizing the emotional toll. “*When the war started, I was about five months pregnant, and my husband was drafted immediately. At first, I watched everything on TV, but it affected me badly. Then I decided I had a baby inside me and needed to protect her, so I disconnected from the news”* [15].

*“*I was glued to war, related videos, and news, and couldn't function. I wasn't sleeping, barely eating, and felt my milk supply was dropping. What helped most was deciding to completely disconnect from the news, because my current role is to be a mother and to do whatever it took to function” [8].

#### Subtheme 2. Internal resources: Motherhood role

The role of motherhood served as an internal resource during the war. Mothers described their love and commitment to their infants as a source of emotional strength, helping them remain focused and grounded. Caring for the infant offered a sense of purpose and stability, while distracting from external chaos. “*Caring for my infant really distracted me. I'm with her most of the time and try to stay positive. My love for her is what strengthens me and gives me energy”* [3].

*“*When I came home, I felt alone, as if all the burden was on me, and it was hard. I tried to stay connected to her; my love for her really helped distract me from the difficulty, and when I was with her, I felt stronger” [7].

Breastfeeding was also a meaningful anchor and stabilizing force, deepening the mother-infant connection, promoting a sense of calm, and enabling emotional regulation during periods of isolation and stress. In the absence of spousal support, breastfeeding provided a sense of control, helping mothers reduce anxiety, regulate emotions, and reaffirm their caregiving role. “*Breastfeeding helped me stay calm …* *I needed to stay relaxed for my infant so I'd be able to produce milk. That kept me grounded”* [15].

“The war, the stress, and the fact that my husband wasn't with me strengthened my desire to protect my infant, to be her anchor and truly bond with her. I think breastfeeding really helped me with that” [17].

“When I was alone and my husband wasn't with me, breastfeeding would help me maintain my bond with my infant and stay calm for her, even when I felt emotionally overwhelmed” [18].

#### Subtheme 3. External resources: support network—the gap between desired and received support

Mothers described receiving support primarily from close family members, particularly from their own mothers, and from friends and professionals. These sources provided emotional reassurance and practical help, which were essential. However, several mothers mentioned gaps in their support networks. The absence of their partners and shared decision-making, and in some cases a lack of involvement from friends or professionals, left them feeling overlooked and burdened. The discrepancy between the support they hoped for and the help they received deepened their sense of need. “*I would have been emotionally stronger if my husband had been with me. I would have had someone to share with, lean on, and make decisions together with. I would have felt calmer and more secure”* [9].

The mothers desired practical help and emotional partnership, with some reflecting on what their partners’ presence could have contributed, had they not been drafted. “*If my husband hadn't been drafted, he would have been a 100% involved father. Caring for my infant is therapeutic for him. When he's around, I feel calmer, happier, and more able to take care of myself”* [12].

The absence of this support was felt not only as a practical loss but as an emotional void that shaped the early parenting experience. However, even limited contact, such as phone calls with words of encouragement, was a source of strength, helping the mothers cope and function in their caregiving role. “*My husband encouraged me from afar. His role allowed me to call him occasionally, and that gave me the strength to function”* [13].

“There were times he manages to be in touch with me by phone, which really helps me function and care for my infant” [2].

Support from family, particularly from the mothers' mothers and sisters, was described as a key external resource that provided emotional comfort and practical assistance. These relationships helped reduce feelings of helplessness and isolation and offered a feeling of protection and buffer against uncertainty. “*I felt like I needed my mother. I wanted to be her little girl again and be protected. I even moved in with her so she could be with me and protect me”.* [11].

“My family is very supportive, especially my mother, who often comes to help. This gives me a sense of security and protection, knowing that I have someone to lean on during this time” [16].

For many, the return to the maternal home symbolized safety and a temporary change in roles, facilitating mothers to be cared for while caring for their own infant. Family members provided hands-on support that made the daily demands of caregiving more manageable. “*I couldn't do anything, not shower, not eat, it was all about him. Luckily, my mom was with me a lot. She and my sister would buy toys and play with him. My family is my main support”*. [19].

Community initiatives helped with meals, childcare, and organized support through local networks.

“I have a supportive community that offers meals and babysitting when needed. They post in a group and whoever needs help gets it … I'm really grateful” [18].

Peer support groups of mothers with partners who were called up were helpful in validating their experiences and reducing feelings of loneliness. “*I feel like people around us don't always understand us mothers. We need more empathy. I spend time with moms who have babies the same age, and it gives me strength.”* [13].

“I joined a group of moms I met after giving birth. We supported each other. I also had a close friend whose husband is a soldier, like mine, and I felt comfortable with her. We went through similar things together” [15].

Mothers' support groups, online and in person, and social media groups, offered mothers a sense of belonging and mutual understanding. Digital platforms afforded access to support and information. “*I joined an [online] group for breastfeeding mothers, and it helped me a lot, truly helped. I mostly read questions from other mothers and asked for advice myself.”* [4].

*“*I participated in online forums for partners of reservists, where they organized [online] meetings with content that really helped me cope with the situation. In one session, we were asked to write a letter to our husbands describing what we were experiencing, and it helped me tremendously” [7].

Mothers reported mixed experiences with professional support, particularly from community nurses and midwives at the maternal infant clinics. These clinics serve as the primary point of contact for mothers after discharge, providing follow-up care, health promotion, and support for emotional adjustment during the postpartum period. Some mothers described compassionate and responsive care that addressed both practical and emotional needs. In these cases, the nurses were perceived as central figures in promoting maternal confidence and trust, providing guidance, reassurance, and continuity of care. Their supportive presence was often remembered as a key protective factor that helped mothers navigate the chaos of wartime motherhood.

“At the maternal-infant clinic, they helped me with breastfeeding … The nurses were amazing, they asked how I was and showed real understanding of my situation.” [15] “I came to the maternal-infant clinic without an appointment, crying. I was worried my infant wasn't gaining weight. The nurse reassured me, weighed him, and the next day, they sent a lactation consultant to my parents’ home. She helped me latch him properly, and from that point, breastfeeding improved.” [13] “At the hospital, the midwife was very supportive; she helped me a lot and guided the baby to latch, so I was able to breastfeed right from the start and had a really positive experience ]8].”

In contrast, some mothers reported that care at the maternal-infant clinics felt limited, technical, and emotionally distant. They described brief encounters focused solely on physical growth measures or documentation, with little inquiry into maternal well-being, leaving them to feel unsupported.‏

“At the maternal-infant clinic, I didn't get much support … I should have received a home visit since it was my first child and she was premature, but no one came … I was struggling” [10]. “The nurse at the maternal-infant clinic shouldn't only care about growth charts. She should ask why I come alone, how I'm coping without my husband … ” [19] “At the maternal-child health clinic, I felt discouraged from continuing to breastfeed. The nurse's comments about my baby's weight really stressed me and undermined my confidence, which was very disappointing” [8].

This theme reflects secondary appraisal and coping, as mothers evaluated which resources were available, reliable, and emotionally meaningful under wartime conditions. Internal resources included maternal commitment, emotional regulation, breastfeeding, and the ability to limit exposure to distressing news. External resources included partner contact when available, family support, peer groups, community initiatives, and professional care. Importantly, coping was not always sufficient or stable; participants' accounts show that resources could buffer distress, but their absence or inconsistency also intensified feelings of isolation and overload.

## Discussion

The current study contributes context-specific qualitative evidence from Israel during the Iron Swords war, showing how wartime threat, partner absence due to reserve or security-force mobilization, disrupted routines, and uneven access to support shaped first-time mothers' postpartum stress, coping, and engagement with care systems. The study invites reflection on what it means to provide nursing care in the context of collective trauma. Nursing, in such circumstances, becomes both a clinical and a moral act, an encounter between human vulnerability and professional compassion. In this section, we discuss each of the themes that emerged from the study and examine their significance in light of the existing literature and the broader context.

### Forced single parenthood

The theme of forced single parenthood brings together mothers' accounts of shattered dreams, loneliness and helplessness, survival parenting, and self-blame. These subthemes show that partner absence was experienced not merely as a practical lack of assistance, but as a disruption of the expected transition to shared parenthood and as a source of emotional vulnerability during the postpartum period. Although the mothers were in committed relationships, the sudden and prolonged absence of the father transformed their experience into forced single motherhood, without the time to prepare, adjust, or build alternative support systems. This role incongruity emerged as a key source of distress, characterized by shattered expectations, loneliness, and emotional overload. These findings align with the Transactional Model (12), in which the primary appraisal of the situation, unexpectedly caring for an infant alone during wartime, is perceived as a major threat to emotional and physical well-being. Partner's absence during a highly vulnerable period, when shared caregiving and emotional presence were expected, created a gap between mothers' internalized ideals of family life and their lived reality. This incongruence often triggered feelings of grief, helplessness, and guilt, in line with previous studies highlighting the mental health risks of disrupted postpartum support due to military assignment during wartime (30, 31).

The concept of “survival parenting” that emerged from participants' narratives echoes prior literature on maternal functioning in contexts of crisis and trauma (13). Mothers described themselves as operating on “autopilot”, attending to their infant's needs while emotionally detached, a pattern that may be understood as an adaptive, yet psychologically costly, coping strategy. The pressure to perform caregiving duties without emotional reciprocity or shared responsibility was compounded by social expectations of “good motherhood”, which further intensified self-blame when ideal standards could not be met (7).

### Complex role of motherhood in war

The theme of the complex role of motherhood in war brings together mothers' accounts of maternal burnout vs. empowerment, role reversal, success vs. failure, and difficulties in emotional bonding alongside the desire to feel normal. Together, these subthemes show how wartime motherhood expanded beyond infant care and required mothers to preserve family functioning, protect the infant, manage their own distress, and, in some cases, care for an injured or emotionally distressed partner. The war disrupted the expected transition to motherhood, placing the entire parenting role on the mother. In some cases, this caregiving responsibility also included caring for a spouse who returned physically and/or emotionally wounded. This dual role heightened mothers’ feelings of exhaustion, invisibility, and emotional conflict, leading to maternal burnout and blurred maternal identity.‏ These findings are consistent with the literature describing role overload and role conflict as significant stressors in maternal mental health (32). Mothers had to care for their infants while also managing their own emotional well-being, shielding their child from the trauma of war, and, when necessary, caring for their partner. The present findings resemble previous descriptions of parental burnout as involving emotional exhaustion, contrast with one's previous parental self, and reduced emotional availability, while showing how these processes may be intensified when first-time motherhood occurs under conditions of war and partner absence (33).

The tension between *burnout and empowerment*, described by participants, can be interpreted as reflecting the dynamic nature of maternal identity formation under pressure. On one hand, mothers reported emotional depletion, irritability, and disconnection from their infants, core symptoms of parental burnout (33). On the other hand, some mothers also experienced a growing sense of competence and resilience, especially as they adapted and coped effectively over time. This ambivalence aligns with theories of post-traumatic growth, which suggest that adversity can produce both vulnerability and strength (34). Importantly, mothers' accounts of emotional disconnection, invisibility, and difficulty feeling “normal” also point to the consequences of insufficient emotional support during a highly dependent postpartum period. This connection is consistent with prior research showing that limited postpartum support, particularly under stressful or conflict-related conditions, may increase the risk of postpartum depression and impair maternal-infant bonding (30, 31, 35).

### Fears and anxieties

The theme of fears and anxieties brings together mothers' accounts of anxious and overprotective mothering, fear for the father, and war-triggered nightmares. These subthemes show that fear was not limited to a general sense of wartime insecurity but entered mothers' daily caregiving decisions, sleep, mobility, and emotional availability. Mothers in this study described a persistent state of hypervigilance rooted in fear for their infant's safety, concern for their partner's life, and anxiety about their own ability to cope. As the war persisted, these stressors were experienced as ongoing psychological burdens, characterized by disrupted sleep and heightened anxiety, consistent with findings among adults in conflict-affected regions (36). Similar to prior studies on conflict-related distress, the mothers in the present study described constant alertness, intrusive worry, and difficulty maintaining ordinary routines under conditions of repeated threat. Sleep disturbance impairs postpartum recovery, further exacerbating anxiety and burnout (37), and war-related distress has been associated with poor maternal sleep and consequent disruptions to infant sleep patterns (11, 36).

Fear for the absent partner emerged as a distinct form of *anticipatory grief*, a phenomenon well-documented among families of deployed military personnel (30). Mothers in this context experienced elevated anxiety and depressive symptoms rooted in ongoing concern for their spouse's safety and in the uncertainty surrounding his return from military service (23, 38, 39). A salient manifestation of this anxiety was the persistent fear of the “knock on the door,” a metaphor for the anticipated notification of a partner's death (40). This fear, described as constant and intrusive, significantly impaired daily functioning and was linked in participants' accounts to sleep disturbance, emotional preoccupation, and difficulty feeling secure in everyday life (39, 41). Such preoccupation with potential loss may hinder maternal-infant bonding, particularly when emotional resources are diverted toward managing war-related distress (11).

### Coping strategies and resources

The theme of coping strategies and resources brings together mothers' accounts of inner strength and resilience, motherhood as an internal resource, breastfeeding as a source of bonding and stability, and external support from partners, family, community, peers, social media groups, and professionals. Together, these subthemes show that coping was not a single strategy, but a dynamic process in which mothers drew on personal, relational, community, and professional resources to maintain caregiving and emotional functioning during wartime.

Internal resources were primarily described by mothers as protective and enabling. These included emotional regulation and emotional work, referring to efforts to adjust emotions to meet social or personal expectations (42–44). For many participants, emotional regulation, self-reliance, love for the infant, commitment to caregiving, and breastfeeding helped create a sense of continuity, bonding, and maternal agency amid chaos. These findings are consistent with the literature describing emotion regulation and meaning-focused coping as important resources in stressful circumstances (12, 45). At the same time, some forms of emotional regulation, such as suppressing distress or “wearing a mask” in front of the infant, may have been adaptive in the short term but emotionally costly when sustained over time.

Some mothers employed problem-focused strategies, such as avoiding exposure to war-related media and restructuring their daily routines to regain a sense of control. External resources, especially support from family, community, and digital networks, played a crucial role in buffering stress (12). Family support offered practical assistance and emotional protection, peer groups provided normalization and shared understanding, and community initiatives helped reduce the burden of parenting alone. These findings correspond with previous research showing that social and community support can buffer stress and strengthen coping during crisis and displacement contexts.

Many mothers emphasized the importance of practical assistance, emotional validation, and professional support, yet described a significant gap between the help they needed and what they received. This variability in care, from empathetic, relational encounters to procedural, task-oriented interactions, highlights the need for attentive, continuous maternal care and relational practices in maternal health. Compassionate and continuous care enabled nurses and midwives to address both physical and emotional needs, fostering a sense of safety and stability in the face of uncertainty. In contrast, fragmented or solely task-focused care left mothers feeling unseen and unsupported, revealing missed opportunities to integrate holistic and psychosocial approaches into routine practice. These accounts underscore that nursing extends beyond clinical care into the moral and emotional domains of human experience, encompassing presence, empathy, and shared vulnerability (46, 47).

Peer support, both online and in-person, also emerged as a valuable coping resource, offering a sense of belonging, normalization, and shared learning experiences (20). However, constant exposure to distressing war-related content on social media could intensify anxiety (3, 48), indicating that digital spaces may both support and strain maternal coping during conflict.

### Study limitations and suggestions for future research

This study has several limitations. Participants' narratives may have been shaped by recall bias or influenced by their current emotional state. Although the qualitative design allowed for rich, in-depth accounts, it was not intended to determine causal relationships or to capture the full range of experiences among all mothers affected by wartime conditions. Because the sample focused exclusively on first-time mothers with absent partners, the findings reflect context-bound insights rather than broader population patterns. In addition, the sample was relatively small and homogeneous in terms of life stage, family status, and educational background, with most participants having an academic education. This may have limited the range of perspectives represented in the study. Recruitment through social media groups and snowball sampling may also have selected mothers who were more socially connected, digitally engaged, and able to access informal support networks, while the experiences of less connected, less resourced, or more socially isolated mothers may be underrepresented. Moreover, because the interviews were conducted online or by telephone rather than face-to-face, the researchers were limited in their ability to observe participants' non-verbal expressions, body language, and contextual cues. This may have limited access to some non-verbal or contextual dimensions of participants’ experiences that might have been more visible in face-to-face interviews.

Future research would benefit from longitudinal designs to explore the long-term effects of wartime postpartum experiences on maternal mental health, family functioning, and child development. Including fathers' perspectives, or conducting dyadic or family-centered qualitative studies, could further illuminate the relational dynamics of parenting under conditions of conflict. As with most qualitative studies, the findings are not intended to be generalizable but to provide in-depth insight into mothers' lived experiences.

### Implications for practice

This study highlights the need to recognize first-time mothers with absent partners as a particularly vulnerable group during armed conflict. At the same time, recommendations for postpartum care in wartime must acknowledge that armed conflict may weaken the very health-system capacity required to provide continuous, relational, and trauma-informed care. Workforce shortages, population displacement, disrupted routines, competing acute-care demands, and limited availability of home-based or mental-health services may constrain what can realistically be implemented.

Healthcare providers, especially community nurses and midwives, should integrate trauma-informed principles into postpartum care and routinely assess maternal mental health alongside physical well-being. However, in conflict settings, these recommendations must be balanced against the reality that health-system capacity may be reduced by workforce shortages, population displacement, competing acute-care demands, and limited availability of home-based or mental-health services (49, 50). Therefore, trauma-informed postpartum care should be implemented through a feasible, tiered approach. At the universal level, even brief postpartum encounters can include emotional acknowledgment, respectful communication, and one or two focused questions about maternal distress, safety, and available support.

Strengthening collaboration between community and hospital-based services is essential to ensure continuity, accessibility, and emotional safety in care. Developing clear referral pathways between maternity wards, maternal-infant clinics, and mental health services can improve timely support for mothers experiencing distress. At the targeted level, mothers with additional risk factors, such as first-time motherhood, partner mobilization, prematurity, evacuation or temporary relocation, breastfeeding difficulties, lack of protected space at home, or signs of emotional distress, should be prioritized for proactive follow-up, referral, or additional support.

Maternal-infant clinics should offer flexible, relational models of support that reduce isolation and accommodate the demands of parenting alone in conflict settings. Rather than recommending resource-intensive services universally, integration with mental health professionals, scheduled follow-up calls, and home visits should be prioritized based on clinical and psychosocial risk. For mothers who are not identified as high risk, lower-intensity support, such as brief check-ins, digital guidance, peer support groups, and clear referral pathways, may be more feasible during periods of conflict-related system strain.

The findings regarding rushed, task-oriented clinic encounters and the missed home visit for a first-time mother of a premature infant should therefore be interpreted not only as gaps in individual provider sensitivity, but also as possible signs of a maternal-care system operating under conflict-related pressure. These recommendations are consistent with humanitarian sexual and reproductive health frameworks, including the Minimum Initial Service Package and the Inter-Agency Field Manual on Reproductive Health in Humanitarian Settings (51), which emphasize prioritizing essential services when capacity is degraded. They also align with the WHO quality-of-care framework for maternal and newborn health (6), which emphasizes both evidence-based clinical care and the experience of care, including respect, communication, emotional support, and dignity.

## Conclusions

The findings illustrate how wartime conditions fundamentally reshape the postpartum experience, intensifying vulnerability while also activating meaningful resilience among first-time mothers. Consistent with the Transactional Model of Stress and Coping (12), mothers' narratives reflected heightened emotional strain, disrupted family roles, and the need to navigate early parenthood without partner support. At the same time, mothers engaged in diverse coping strategies that helped them manage uncertainty and maintain caregiving continuity. These insights underscore the importance of integrating emotional sensitivity and trauma-informed, relational approaches into postpartum care, ensuring that maternal health services address both psychological and relational needs during periods of conflict and other large-scale crises.

## Data Availability

The datasets presented in this article are not readily available because The datasets generated and analyzed during the current qualitative study are not publicly available due to ethical, confidentiality, and methodological considerations inherent to qualitative research. Data sharing is restricted in accordance with the approval of the institutional ethics committee and the informed consent provided by the participants. Requests to access the datasets should be directed to yifat findling, yifat.f12@gmail.com.
