# Supplementary material for: First-time postpartum motherhood during armed conflict: experiences, challenges, coping, and support needs among women with mobilized partners
Source: Front Glob Womens Health. 2026 Jul 16;7:1774107. doi: 10.3389/fgwh.2026.1774107 (PMC13422384; doi:10.3389/fgwh.2026.1774107)
Supplement: Supplementary file 1 [file Table1.docx]

| Question Number | Interview Question |
| --- | --- |
| 1 | Please tell me about yourself: your age, marital status, occupation, education, and your child’s age. |
| 2 | Tell me about your pregnancy and birth experience. Was your partner present? If not, who accompanied you? How did this affect you and your partner? When did they first meet your infant? |
| 3 | Tell me about your experience as a new mother during the war, while your partner was mobilized. How did you experience this period? What emotions did you feel? |
| 4 | Were you able to breastfeed? If so, for how long? Did you experience any difficulties while breastfeeding? |
| 5 | Were you given the option to use a breast milk bank? What are your thoughts on this? How did the war and your partner’s absence affect your breastfeeding experience? |
| 6 | To what extent did the healthcare staff support you in dealing with breastfeeding challenges? Was the guidance provided online? Did you feel that support was accessible and available? Did you receive support through social media? |
| 7 | Describe the impact of the security situation on your and your baby’s sleep routine. Did you experience sleep difficulties? |
| 8 | Do you believe your sleep difficulties affected your baby’s sleep? How? |
| 9 | How do you cope with the daily challenges of caring for your baby on your own? What helps you manage? |
| 10 | How do you deal with the concern for your partner while also caring for your baby? How does this affect your baby and your functioning as a mother? |
| 11 | How do you feel about your ability to meet your baby’s needs during this time? Do you notice any changes in your relationship? |
| 12 | What role do the community, extended family, and friends play during this time? Has your support system changed? |
| 13 | Tell me about moments when you felt exhaustion or burnout as a mother. How did this manifest? How did you cope? |
| 14 | What do social media mean to you during this time? How do they affect your experience of motherhood? |
| 15 | What would you want professionals in healthcare, social welfare, or security to know about your experience as a young mother during wartime? Looking back, how has this period influenced your development as a mother? What have you learned about yourself? What do you do to promote your own well-being? What do you wish for yourself moving forward? |
| 16 | Is there anything else you would like to add? |
